# Factors associated with healthcare-seeking behavior for symptomatic acute respiratory infection among children in East Africa: a cross-sectional study

**DOI:** 10.1186/s12887-022-03680-w

**Published:** 2022-11-15

**Authors:** Dagmawi Chilot, Kegnie Shitu, Yibeltal Yismaw Gela, Mihret Getnet, Bezawit Mulat, Mengistie Diress, Daniel Gashaneh Belay

**Affiliations:** 1 College of Health Sciences, Center for Innovative Drug Development and Therapeutic Trials for Africa (CDT-Africa), Addis Ababa University, Addis Ababa, Ethiopia; 2 Department of Human Physiology, College of Medicine and Health Science, School of Medicine, University of Gondar, P.O. Box 196, , Gondar, Ethiopia; 3 Department of Health Education and Behavioral Science, College of Medicine and Health Science, University of Gondar, Institute of Public Health, Gondar, Ethiopia; 4 Department of Human Anatomy, College of Medicine and Health Science, School of Medicine, University of Gondar, Gondar, Ethiopia

**Keywords:** Healthcare-seeking behavior, Acute respiratory infection (ARI), Children, Women, Guardians, Caregivers, Medical care, East Africa

## Abstract

**Background:**

Although there has been promising progress in the reduction of child mortality from ARI, the magnitude is high yet, especially in East Africa. Since mothers/guardians decide upon the type and frequency of healthcare services for children, their good healthcare-seeking behavior could prevent acute respiratory infections (ARI) related mortality and morbidity. This study aimed to investigate the pooled prevalence and factors associated with healthcare-seeking behavior of children younger than five years with ARI symptoms by using data from nationally representative surveys of East Africa.

**Methods:**

We analyzed secondary data based on the eleven East African Demographic and Health Survey data. Both Individual and community level variables were considered for this study and a multilevel binary logistic regression model was fitted to identify associated factors of children’s healthcare-seeking behavior for ARI symptoms. STATA V.14 software was used to clean, recode and analyze the data. All variables with a *p*-value = 0.2 in the bi-variable analysis were considered for the multivariable multilevel analysis. Adjusted OR (AOR) with 95% CI was reported to reveal significantly associated factors in the multivariable multilevel analysis.

**Result:**

The overall prevalence of healthcare-seeking behavior of under-five children for ARI symptoms was 64.4% in East Africa. In the multilevel analysis, the following characteristics were found to be the most important factors of children healthcare seeking behavior for ARI symptoms (*P* < 0.05): Rural residence [AOR = 0.51, 95% CI (0.37–0.65)], high community level media usage [AOR = 1.63, 95% CI (1.49–1.79)], high community level women education [AOR = 1.51, 95% CI (1.39–1.66)], primary education [AOR = 1.62, 95% CI (1.45–1.82)], secondary education and above [AOR = 1.99, 95% CI (1.71–2.32)], working mother [AOR = 1.33, 95% CI (1.20–1.48)], unmarried women [AOR = 1.15, 95% CI (1.04–1.27)], media access [AOR = 1.43, 95% CI (1.20–1.58)], richest [AOR = 1.39, 95% CI (1.29–1.51)], distance to health facility not a big problem [AOR = 1.11, 95% CI (1.02–1.21)], Place of delivery at health facilities [AOR = 1.77, 95% CI (1.60–1.95)], age of child 7–23 months [AOR = 1.59, 95% CI (1.39–1.82)], age of child 24–59 months [AOR = 1.24, 95% CI (1.09–1.41)] in comparison with children aged 0–6 months, family size > 10 [AOR = 1.53, 95% CI (1.22–1.92)].

**Conclusions and recommendations:**

The overall prevalence of children’s healthcare-seeking behavior for ARI symptoms was found relatively low in East Africa, ARI symptoms were determined by individual-level variables and community-level factors. Targeted interventions are needed to improve socioeconomic and health systems to overcome the problem of acute respiratory infection in children. Special attention is required to empower local health staff and health facilities to provide proper diagnosis and management of ARI cases in East Africa.

## Background

Acute respiratory infection (ARI) is one of the most frequent childhood diseases that remains a huge public health problem globally [1–4]. Sub-Saharan Africa (SSA) is the region with the highest child mortality in the world [5–8]. Meanwhile, one of the targets of the third Sustainable Development Goal (SDG 3) is ending preventable neonatal and under-five deaths by the year 2030 [9, 10]. The World Health Organization (WHO) estimates that seeking prompt and appropriate care could reduce child deaths due to ARI by 20% [11]. Since mothers decide upon the type and frequency of healthcare services for children, their good healthcare-seeking behavior could prevent ARI-related mortality and morbidity. Previous studies reported that delay in seeking appropriate care and not seeking any care contributes to a large number of child deaths from ARI [12–16].

Generally, the prevalence of seeking out healthcare for mothers of their children is low in East Africa [17, 18]. In this region, the lack of child healthcare services has been a barrier to accessing and seeking healthcare for children with ARI symptoms. Another problem was poor knowledge of mothers to identify danger signs or appropriate treatment to be given to their children for ARI [15, 19, 20]. Previous country-specific studies have revealed that socio-demographic, economic, healthcare service, and household characteristics were major factors in seeking healthcare for children. In addition, perceived illness severity and the mother’s recognition of certain signs and symptoms of childhood illness were critical factors in seeking prompt healthcare for ARI symptoms [21–24].

However, those country-specific studies couldn’t provide a panoramic view of the healthcare-seeking behavior for ARI symptoms in East Africa. Therefore, assessing the prevalence and determinants of seeking healthcare based on pooled nationally representative data will give insight to health professionals and policymakers to understand the burden, fight the problem together and set possible interventions at both individual and community levels. To improve healthcare-seeking behavior, an intervention of regional and international stakeholders to the major determinants is needed and the findings of this study could help to design evidence-based public health decisions. Moreover, this study was a pooled analysis that could increase the study power to permit a full examination of effect modification within the data.

Given these and to improve the healthcare-seeking behavior of children in East Africa, it is essential to expand our understanding of the current prevalence, and determinants associated with the problem. Thus the purpose of our study was to assess the prevalence and factors associated with healthcare-seeking behavior for symptomatic acute respiratory infection among children in East Africa.

## Materials and methods

### Study design, setting, and period

The Demographic and Health Surveys (DHS) used a cross-sectional survey study design to collect the data. The study was conducted in eleven East African countries from 2012 to 2020. We included data from 11 countries [(Burundi (2016/2017), Comoros (2012), Ethiopia (2016), Kenya (2014), Malawi (2015/2016), Mozambique (2015), Rwanda (2019/2020), Tanzania (2015/2016), Uganda (2016), Zambia (2018), and Zimbabwe (2015)] that submitted publically available datasets. These years indicate the most recent DHS data for each country, and those countries that have two years listed came from one dataset.

### Data source

We pooled the data from the DHSs conducted in eleven East African countries. The DHS is a nationally representative survey that is conducted in low- and middle-income countries globally. The DHS survey used a stratified two-stage sampling technique in each country. First, enumeration areas (EAs) were selected randomly from the sampling frame. In the second stage, a sample of households is drawn from a list of households in each EA selected. The data in this file were captured from women who are either caregivers of children under five or gave birth within the five years preceding the surveys. Multi-country analysis using DHS survey data is reasonable as the surveys follow the same questionnaires, sampling procedure, data collection, and coding [25].

For this analysis, we used the children under age 5 recode (KR) file and we included only children under age 5 with symptoms of ARI at any time in the 2 weeks preceding each survey. In DHS surveys the sample is selected with unequal probability to increase cases available for certain areas for which statistics are needed. Therefore, we weighted the sample using the individual weight of women (v005) to produce the proper representation. Hence sample weights were generated by dividing (v005) by 1,000,000. The survey year and the weighted sample were taken from each country indicated in Table 1.

## Population

Our source population was all under-five children who lived in East Africa from 2012 to 2020. The study population was all under-five children who lived in East Africa, had ARI symptoms, and were alive. Sample weight (v005/1,000,000) was used to correct for over- and under-sampling and generalizability of the findings.

**Table 1:** Distribution of the study sample and year of a survey by the country

Country	Survey year	Sample used	% of the sample used
Burundi	2016/2017	1,989	14.35
Comoros	2012	203	1.47
Ethiopia	2016	1,265	9.12
Kenya	2014	3,170	22.87
Malawi	2015/2016	1,947	14.05
Mozambique	2015	737	5.31
Rwanda	2019/2020	695	5.01
Tanzania	2015/2016	575	4.15
Uganda	2016	2,427	17.51
Zambia	2018	286	2.06
Zimbabwe	2015	567	4.09
**Total**		13,861	100

## Definition of variables

### Outcome variable

The DHS of the selected East African countries asked mothers/ caregivers to report whether their children had been ill with symptoms of cough accompanied by short and rapid breathing in the two weeks before the survey. They were also asked if they had sought treatment and where the treatment had been sought. The outcome variable of this study was healthcare-seeking behavior for children under age five with ARI symptoms. According to the DHS data seeking medical care refers to the number of living children under age 5 with symptoms of ARI in the 2 weeks preceding the survey for whom treatment was sought excluding advice or treatment from a traditional practitioner. It was coded as “1” if the mother/caregiver “sought medical care” in public health care facilities or at private health care facilities and”0" otherwise. In this study, healthcare seeking was operationally defined as a mother/caregiver seeking an expert opinion or treatment from public or private health facilities for a child who shows symptoms of ARI [26].

### Independent variables

Major explanatory variables which are logically related and have < 5% missing value were included in the analysis. Those variables were considered on two levels. Level-I included individual socio-demographic, economic, and behavioral factors. Those factors were maternal age, maternal education, maternal occupation, marital status, family size, media exposure, wealth index, age of the child, sex of the child, distance to health facilities, place of delivery, sex of household head birth order, health insurance status, and twin status. For some variables, categorization was done for comparison purposes with previous research.

Level II was community-level factors and was done to realize whether cluster-level variables affected the healthcare-seeking behavior of children. To generate community-level variables (community media exposure, community poverty, and community women’s education) we did an aggregation of individual-level variables at the cluster level and categorized them as higher or lower based on 50th percentile. Residence, which is a direct community-level variable was used without any manipulation.

Community-level media usage refers to the proportion of women in the community who used radio, TV, and newsletter and it was categorized as low community-level media usage and high community-level media usage. “Low” refers to communities in which < 50% of respondents had media access while “high” indicates communities in which = 50% of respondents had media access.

Community level poverty refers to the proportion of women in the community who had low wealth quintiles (poorest and poorer). It was categorized as low if the proportion of low wealth quintile households was < 50% and high if the proportion was = 50%.

Community-level women’s education refers to the proportion of women in the community who had formal education (primary and above). It was categorized as low if communities in which < 50% of respondents had formal education and high if = 50% of respondents had attended formal education [27].

## Statistical analyses

The datasets were extracted from each of the 11 country’s data files. STATA version 14.2 was used to clean, recode and analyze the data. Then we append the dataset of each country to generate pooled data. A multilevel random intercept logistic regression model was carried out to estimate the influence of individual-level and community-level variables and the likelihood of seeking treatment for ARI symptoms. Four models were constructed and they comprised the null model (model 0) without any explanatory variables, Model I with individual independent variables only, Model II with community-level factors only, and Model III with both individual-level and community-level variables.

Since the models were nested (one variable is nested within the other) we compared them using deviance (- 2 log-likelihood). Intra-cluster correlation coefficient (ICC) was done to measure the variation of healthcare-seeking behavior between clusters. We also calculated median odds ratio (MOR) and proportional change in variance (PCV) to determine whether clustering occurred or not and compare models respectively. All variables with a *p*-value = 0.2 in the bi-variable analysis were fitted in the multivariable model. Variables at both community and individual levels were presented as odds ratios with a 95% CI. In the adjusted model, a *P* value < 0.05 was used to declare it statistically significant.

## Results

### Socio-demographic, economic, and health service-related characteristics of respondents

A total of 13,861 women/caregivers of children under five or who gave birth within the five years preceding the surveys were included in the analysis. Of the total, 47.38% were aged 25–34 years. More than one-fifth (21.56%) of mothers/caregivers had no formal education. With regard to the wealth index, almost half (46.68%) were poor. About 88.55% of women/caregivers were uninsured and 67.11% had media access (Table 2).

**Table 2:** Socio-demographic, economic, and health service-related characteristics of respondents

Variables	Categories	Unweighted frequency (%)	Weighted frequency (%)
Age of mothers	15–24	4,213 (31.62)	4,352 (31.40)
	25–34	6,255 (46.95)	6,568 (47.38)
	35–49	2,854 (21.42)	2,941 (21.22)
Mothers educational level	No education	2,914 (21.87)	2,989 (21.56)
	Primary education	7,385 (55.43)	7,732 (55.78)
	Secondary and above	3,023 (22.69)	3,140 (22.66)
Mothers occupation	Not working	2,778 (23.92)	3,041 (24.87)
	Working	8,835 (76.08)	9,185 (75.13)
Mothers marital status	Married	8,959 (67.25)	9,273 (66.90)
	Not married	4,363 (32.75)	4,588 (33.10)
Number of a household member	1–4	4,195 (31.49)	4,473 (32.27)
	5–10	8,479 (63.65)	8,738 (63.04)
	> 10	648 (4.86)	650 (4.69)
Media access	No	4,318 (32.43)	4,556 (32.89)
	Yes	8,997 (67.57)	9,296 (67.11)
Wealth index	Poor	6,525 (48.98)	6,470 (46.68)
	Middle	2,513 (18.86)	2,785 (20.09)
	Rich	4,284 (32.16)	4,606 (33.23)
Age of child	0–6 months	1,646 (12.43)	1,707 (12.40)
	7–23 months	4,796 (36.20)	4,982 (36.18)
	24–59 months	6,805 (51.37)	7,080 (51.42)
Sex of child	Male	6,891 (51.73)	7,150 (51.59)
	Female	6,431 (48.27)	6,710 (48.41)
Distance to a health facility	Not big problem	5,880 (54.05)	6,015 (52.30)
	Big problem	4,999 (45.95)	5,487 (47.70)
Place of delivery	Home	3,750 (28.80)	3,943 (29.07)
	Health facilities	9,269 (71.20)	9,621 (70.93)
Sex of household head	Male	9,882 (74.18)	10,415 (75.15)
	Female	3,440 (25.82)	3,445 (24.85)
Birth order	First	3,048 (22.88)	3,198 (23.07)
	2–4	6,505 (48.83)	6,755 (48.74)
	5+	3,769 (28.29)	3,907 (28.19)
Health insurance status	Insured	1,374 (11.82)	1,401 (11.45)
	Uninsured	10,254 (88.18)	10,837 (88.55)
Twin status	Single birth	12,928 (97.04)	13,456 (97.08)
	Multiple births	394 (2.96)	405 (2.92)

### Prevalence of health care seeking behavior of children for ARI symptoms in East Africa

**Fig. 1:**
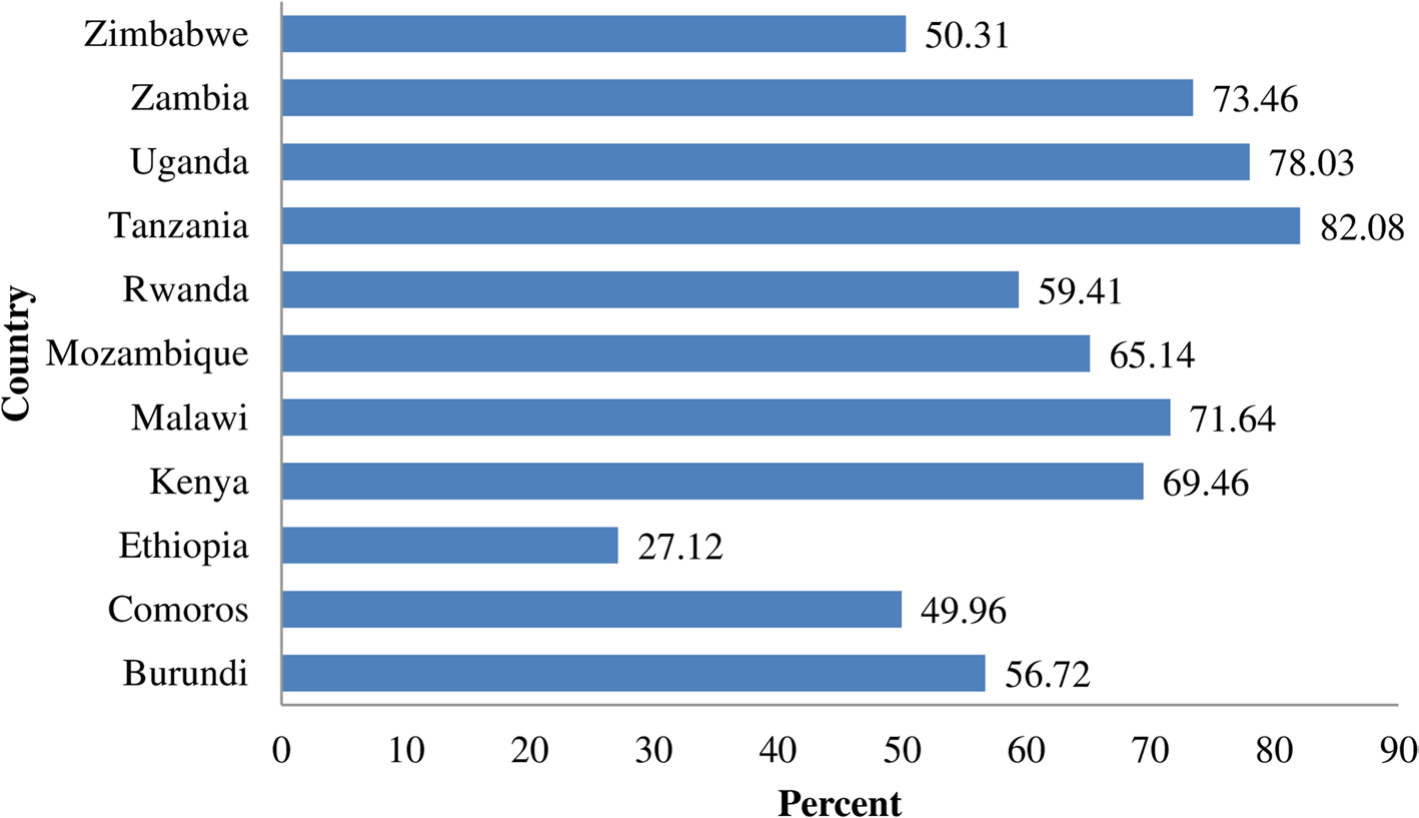
Prevalence of healthcare-seeking behavior of children for ARI symptoms in East Africa

The pooled prevalence of seeking healthcare for ARI in children under 5 years old in East Africa was 64.40% (95% CI; 63.02 - 65.06%). Tanzania (82.08%) had the highest healthcare-seeking behavior and Uganda (78.03%) was in second place. in Ethiopia, children’s healthcare-seeking behavior for ARI symptoms was the least, which was 27.12% (Fig. 1).

### Multilevel logistic regression analysis of healthcare-seeking behavior

In the multilevel analysis, individual-level variables such as maternal education, maternal occupation, marital status, number of household members, media access wealth index, age of the child, distance to health facilities, and place of delivery were significantly associated factors. Regarding community-level factors, residence, community media usage, and community women’s education were found significant associations with healthcare-seeking behavior for ARI symptoms.

Among the factors, maternal education, place of delivery, residence, and community media usage were the most strongly associated variables. The odds of seeking healthcare for children from mothers who had attended primary education and secondary and above were 1.62 and 1.98 times higher than those who are from illiterate mothers [AOR = 1.62; 95% CI; 1.45–1.82] and [AOR = 1.98; 95% CI; 1.70–2.32] respectively. Children who were born in the health facilities were 1.77 times higher in seeking healthcare compared to their counterparts [AOR = 1.77; 95% CI; 1.60–1.95]. Urban residents had higher odds of seeking healthcare than rural residents [AOR = 1.53; 95% CI; 1.40–1.66]. High community media usage was positively associated with seeking healthcare [AOR = 1.63; 95% CI; 1.49–1.79] (Table 3).

**Table 3:** Multivariable multilevel logistic regression analysis results of both individual-level and community-level factors associated with healthcare-seeking behavior in East Africa

Variables	Categories	Null model	Model 1 AOR [95% CI]	Model 2 AOR [95% CI]	Model 3 AOR [95% CI]
Age of mothers	15–24		1.00		1.00
	25–34		0.94 (0.84- 1.06)	–––––––-	0.94 (0.84—1.06)
	35–49		0.97 (0.82- 1.14)	–––––––-	0.96 (0.82—1.14)
Mothers educational level	No education		1.00	–––––––-	1.00
	Primary education		1.63 (1.47- 1.82)***	–––––––-	1.62 (1.45—1.82)***
	Secondary and above		2.01 (1.74- 2.33)***	–––––––-	1.98 (1.70—2.32)***
Mothers occupation	Not working		1.00	–––––––-	1.00
	Working		1.34 (1.20- 1.48)***	–––––––-	1.33 (1.20—1.48)***
Mothers marital status	Married		1.00	–––––––-	1.00
	Not married		1.16 (1.07—1.29)***	–––––––-	1.15 (1.04—1.27)**
Number of a household member	1–4		1.00	–––––––-	1.00
	5–10		1.07 (0.97—1.19)	–––––––-	1.09 (0.99—1.21)
	> 10		1.53 (1.21—1.92)**	–––––––-	1.53 (1.22—1.92)***
Media access	No		1.00	–––––––-	1.00
	Yes		1.46 (1.33—1.61)***	–––––––-	1.43 (1.20—1.58)***
Wealth index	Poor		1.00	–––––––-	1.00
	Middle		0.94 (0.85—1.04)	–––––––-	0.89 (0.79- 1.01)
	Rich		1.37 (1.28—1.47)***	–––––––-	1.39 (1.29—1.51)**
Age of child	0–6		1.00	–––––––-	1.00
	7–23		1.54 (1.34—1.78)***	–––––––-	1.59 (1.39—1.82)***
	24–59		1.09 (0.97—1.22)	–––––––-	1.25 (1.10—1.41)*
Sex of child	Male		1.00	–––––––-	1.00
	Female		0.93 (0.86—1.01)	–––––––-	0.94 (0.87—1.02)
Distance to a health facility	Big problem		1.00	–––––––-	1.00
	Not big problem		1.13 (1.02—1.21)***	–––––––-	1.11 (1.01—1.20)*
Place of delivery	Home		1.00	–––––––-	1.00
	Health facilities		1.72 (1.55—1.90)***	–––––––-	1.77 (1.60—1.95)***
Sex of household head	Male		1.00	–––––––-	1.00
	Female		1.02 (0.92—1.12)	–––––––-	1.01 (0.90—1.11)
Birth order	First		1.00	–––––––-	1.00
	2–4		1.09 (0.96—1.23)	–––––––-	1.08 (0.95—1.22)
	5 +		1.08 (0.91—1.28)	–––––––-	1.06 (0.89—1.26)
Health insurance status	Insured		1.00	–––––––-	1.00
	Uninsured		0.99 (0.87—1.13)	–––––––-	0.97 (0.85—1.11)
Twin status	Single birth		1.00	–––––––-	1.00
	Multiple births		1.22 (0.94—1.58)	–––––––-	1.21 (0.93—1.58)
**Community level variables**					
Residence	Rural		–––––––-	1.00	1.00
	Urban		–––––––-	1.70 (1.65- 1.80)***	1.53 (1.40- 1.66)***
Community-level media usage	Low		–––––––-	1	1.00
	High		–––––––-	1.23 (1.09- 1.39)**	1.63 (1.49 -1.79)**
Community-level women education	Low		–––––––-	1.00	1.00
	High		–––––––-	1.21 (1.07- 1.36)**	1.51 (1.39- 1.66)**
Community poverty	Low		–––––––-	1.00	1.00
	High		–––––––-	1.02 (0.89—1.11)	1.09 (0.94—1.20)
**Random effect**	Variance	0.43	0.42	0.39	0.34
	ICC	0.12	0.11	0.10	0.09
	MOR	1.69	1.66	1.52	1.50
	PCV	Reff	2.38	10.26	26.47
**Model comparison**	Log likelihood ratio	-8795	-6990	-8756	-6988
	Deviance	17,590	13,980	17,512	13,976
	Mean VIF		1.62	1.06	1.60

*ICC* Inter cluster correlation coefficient, *MOR* Median odds ratio, *PCV* proportional change in variance, *AOR* Adjusted odds ratio, *CI* Confidence interval, null model without any explanatory variables, Model I with individual independent variables only, Model II with community-level variables only, and Model III with both individual-level and community-level variables

* = *P*-value < 0.05, ** = *P*-value < 0.01, *** = *P*-value < 0.001

## Discussion

ARIs are a major contributor to child mortality and disease burden among under-five children in East African countries [14, 28–30]. Our study aimed to assess the pooled prevalence and associated factors of healthcare-seeking behavior of children for ARI symptoms in east Africa using the pooled DHS data. With inter-country variations, the overall prevalence of health care seeking was found 64.40% ranging from 27.12% in Ethiopia to 82.08% in Tanzania. This variation could be due to uneven distribution of child healthcare facilities, health policy, economic status, and differences in socio-cultural factors across the countries.

In the multilevel analysis, some individual and community-level factors were significantly associated with the healthcare-seeking behavior of children for ARI. Among the individual level variables, this study revealed a significant association between a mother’s education and healthcare-seeking behavior. It was found that the odds of healthcare-seeking behavior among women who had formal education were higher in East Africa. Other previous studies done in Ethiopia Kenya and Pakistan also support this finding [31–33]. Higher reporting could be explained that educated women have the knowledge to recognize any ARI symptoms and a good perception of health facilities. Moreover, it might be attributed to the reality that education can improve the mother’s/caregiver’s healthcare-seeking behavior for their children with ARI symptoms.

Children of working mothers were found to be better at seeking healthcare for ARI. East Africa is a vast region with different socioeconomic, availability, and affordability of health care facilities. Income was a key player in seeking care in health facilities and women who have a job could generate more money and bring their children to health facilities when he/she showed ARI symptoms [12, 34]. However, working mothers could also be busy because of work to take their children to healthcare facilities [21, 35]. Generally, occupation could affect seeking healthcare positively or negatively.

Women’s/caregivers autonomy in health decision-making is critical to seeking prompt healthcare for their children [36]. Women who are not in a union could have better autonomy in making decisions without the interference of their spouses compared to their counterparts. Our finding indicated that being not married was significantly associated with higher odds of healthcare-seeking behavior. Regarding family size, our study revealed that children living with greater than ten household members were more likely to peruse healthcare for ARI compared with children whose family members were less or equal to ten. However, this study is not in conformity with various works of literature [37, 38]. The characteristics of the household members could determine this result. For example, if the family members are old enough to help, the mother could have time to seek healthcare for her child.

Mothers of a child who had access to media (newspaper, radio, television) were more likely to seek treatment for ARI [38–40]. Mass media can shape mothers’ beliefs, attitudes, and norms, which, in turn, influence healthcare-seeking behaviors and increase mother’s/caregivers awareness regarding the importance and urgency of child healthcare. However, radio and television may not be affordable for the poor. The household wealth index could also affect seeking healthcare because of transportation costs and other related expenses on the way to healthcare facilities. In this study, children from high household economic status were more likely to seek health care than children from low household wealth index status. This finding was consistent with other results from Ethiopia, Tanzania, and Mongolia [37, 41, 42].

Along similar lines, we also identified the age of the child as a significant determinant of healthcare-seeking behavior. Children aged greater than seven months had increased odds of seeking healthcare for ARIs. Previous studies identified the youngest children would be the most to be brought to healthcare facilities if they got sick [41, 43]. However our finding could be true because we have managed chance (using 95% CI), bias (DHS data collected using the same standard tools across countries), and confounding (fitted multivariable analysis).

Poor road infrastructure has been a major problem for East African countries and mothers who reported the distance to reach health facilities as a big problem were less likely to seek healthcare. Sometimes accessing health care is unthinkable because of the costs of travel, very long distances, and lack of transportation [12]. Poor infrastructure could also hinder mothers to give birth at health facilities. Our study has found that women who gave birth in health facilities had a higher likelihood to seek healthcare for their children. This could be explained that those who gave birth at health facilities would take their children to medical centers for post-natal clinics and immunizations. On the way to these services, mothers/caregivers might bring their child for any ARI symptoms for medical care.

The study demonstrated that the inclusion of community-level variables was important in explaining the variations in healthcare-seeking behavior. Community-level variables such as residence, community media usage, and community women’s education showed significant effects. Model III was the best-fitted model since it has the highest log likelihood (-6988) and the lowest deviance (13,976) value. The PCV in model III was 26.47%, meaning that about 26.47% of the total variability in the healthcare-seeking behavior for ARI was explained by the full model.

It has been reported that rural residents have limited access to healthcare facilities, health insurance, and a shortage of healthcare professionals [21, 44–47]. These problems could hamper their healthcare-seeking behavior and our study also established that living in rural areas was associated with lower odds of seeking healthcare. Moreover, it has been reported that in rural areas mothers/caregivers sometimes prefer to treat ARI with homemade medicines/remedies before visiting a health facility [48, 49].

Consistent with previous studies done in different country’s DHS data [28, 38, 40], our finding in East Africa showed that high community level media usage had higher odds of healthcare-seeking behavior when compared with low community media access. This might be explained that whether the householder has television/radio or not, they could receive information from their neighbors/ community about the need for healthcare for ARI symptoms as the community has media access.

Our study also found higher odds of healthcare-seeking behavior for ARI in high community-level women’s education compared with lower women’s community-level education. This finding is in line with other studies [47, 50]. Mothers’ knowledge of the ARI symptoms was an important determinant of care-seeking behavior. Thus the community level of education could have a direct impact on the mother’s/caregivers knowledge and can be exploited to improve their health-seeking behavior for ARI. Mothers/caregivers could get a consultation from the community when their children got sick and this could have a positive association with healthcare-seeking behavior for ARI symptoms.

Our study has some important strengths and limitations. Among the strengths, it was conducted using nationally representative pooled data from 11 East African countries. The large sample size has adequate power to detect the true effect of individual and community level factors. Additionally, we fitted the appropriate model to address the DHS data’s hierarchical nature and sampling weight was applied during the analysis to get reliable estimates. As a limitation, given the cross-sectional nature of the study design, the finding from our study may not establish a causal relationship between independent variables and healthcare-seeking behavior for ARI. Besides, we only included children reported as symptomatic and this could introduce selection bias, perhaps children with mild or paucisymptomatic infections were not identified as symptomatic by their parents and did not participate. Moreover, the difference in years of DHS data collection could compromise the comparison between countries. These differences across countries are due to differences over time rather than true differences between the countries.

## Conclusion

Our study found that mother’s/caregivers seeking healthcare for ARI symptoms were determined by individual-level variables (maternal socioeconomic and household characteristics) and community-level factors (residence, community education, and community media access). Therefore, governments and stalk holders should work in concert at improving socioeconomic and health systems to mitigate the problem of poor access to healthcare for ARIs of children. Educational intervention for mothers/caregivers on the need to seek appropriate medical care in health facilities for ARI symptoms is also recommended. Giving special attention on strengthen local health staff and health facilities could be imperative to provide proper diagnosis and management of ARI cases in East Africa.

## Data Availability

Data are available in a public, open access repository and the data set can be accessed online from www.measuredhs.com/data.

## Abbreviations

AOR: Adjusted Odds Ratio
ARI: Acute Respiratory Infection
DHSs: Demographic and Health Surveys
ICC: Intra cluster correlation coefficient
LRIs: Lower Respiratory Tract Infections
MOR: Median odds ratio
PCV: A proportional change in variance
SDG 3: Third Sustainable Development Goal
SSA: Sub-Saharan Africa
URIs: Upper Respiratory Tract Infections
WHO: World Health Organization
